# Editorial: Cardiotoxicity induced by radiotherapy and/or chemotherapy after cancer treatment

**DOI:** 10.3389/fonc.2022.1087928

**Published:** 2022-11-22

**Authors:** Marjan Boerma, Omid Azimzadeh, Nadia Pasinetti, Virginie Monceau

**Affiliations:** ^1^ University of Arkansas for Medical Sciences, Department of Pharmaceutical Sciences, Little Rock, AR, United States; ^2^ Section Radiation Biology, Federal Office for Radiation Protection (BfS), Neuherberg, Germany; ^3^ Department of Radiation Oncology, University of Brescia, Brescia, Italy; ^4^ Laboratory of Radiotoxicology and Radiobiology, Institute for Radiation Protection and Nuclear Safety (IRSN), Fontenay-Aux-Roses, France

**Keywords:** chemotherapy, targeted therapy, cardiotoxicity, radiotherapy, cancer treatment

## Introduction

It is our privilege to present 11 articles inthis Frontiers Oncology Research Topic on “*cardiotoxicity induced by radiotherapy and/or chemotherapy after cancer treatment*”. The therapeutic management of cancer has progressed over the last 10 years and has led to a significant increase in patient survival rates. Radiotherapy (RT) is classically applied in multiple fractions administered over several weeks to kill cancer while sparing normal tissue as much as possible. However, the corollary is the development of delayed toxicities that alter the quality of life of patients. Chemo- and/or RT-induced cardiovascular disease (CVD) is recognized as a worrisome side effect in patients with thoracic cancers.

This toxicity appears to be amplified if there are comorbidities as presented in the article by Banfill et al. that shows the effect of radiation dose on cardiac death after thoracic RT is different in patients with and without cardiac comorbidities. Therefore, CVD risk factors should be identified and managed in conjunction with RT for lung cancer. In the same context, Mirestean et al. describe the concerns that should be considered in breast cancer (BC) therapy. BC is the most common cancer in women worldwide, often treated with RT including whole breast irradiation (WBI). Recent data recommend the use of hypofractionation (HF)-WBI regardless of age or stage of disease. However, some studies reported an increased incidence of cardiac death with HF-WBI, particularly in patients with pre-existing cardiac risk factors at the time of treatment. There is also a need to develop multivariable radiobiologic models including histologic, molecular, and clinical parameters to identify risk groups and dosimetric tolerance to limit the incidence of late cardiac events. Abravan et al. describe the use of real-world data (RWD) to study radiation-induced heart disease (RIHD) in lung cancer patients, summarizing how heart dosimetric factors associate with outcome, strength, and limitations of the RWD studies, and how RWD can be used to assess a change to cardiac dose constraints. Since RT of BC can result in an increased risk of long-term major CVD events, it is critical to detect subclinical left ventricular (LV) dysfunction early in RT treated BC patients to determine the dose-response relationships between cardiac doses and these events. The results of Locquet et al. highlight that all cardiac doses were strongly associated with the development of subclinical LV dysfunction 6 months after RT. It remains to be determined whether global longitudinal strain measurements at baseline and 6 months after RT can predict subclinical events occurring 24 months after RT. In addition, few studies suggested that RT of BC can induce cardiac arrhythmias and conduction disorders. However, the association between mean heart dose and doses to cardiac substructures is less studied. Errahmani et al. performed an exploratory investigation on BC patients treated with RT, which is the first study suggesting that irradiation of the right atria may require special attention regarding the risk of cardiac arrhythmia and conduction disorders. Regardless of the technique used, during RT, healthy tissues located in the irradiation fields are exposed. This is the main reason for the development of RT techniques in the treatment of left-sided BC, where conventional RT leads to cardiac radiation exposure, such as the deep inspiration breath-hold (DIBH), as described by Lu et al. Whether the DIBH regimen is an optimal solution for left-sided BC remains unclear. This meta-analysis aimed to elucidate the differences between DIBH and free-breathing for patients receiving RT for left-sided BC and provide a practical reference for clinical practice. This study suggests more widely use of DIBH in clinical practice because of its excellent dosimetric performance. An article by Kimpe et al. deals with the social impact of long-term effects of radiation-induced cardiotoxicity as an important concern during the treatment of BC. This study applied health economic modelling techniques to estimate attributed CVD-related costs and disutility. Their analyses suggest that the cost of past investments to achieve the mean heart dose (MHD) in current practice seems justified when considering the gains from cost resulting from radiation-induced CVD events. The question remains to be answered is whether costs associated with further investments in technological advancements offset the expected benefit from reducing the MHD. Altogether, epidemiological and clinical data underline the importance of cardiac side effects after RT, but the pathophysiological, cellular and molecular bases of such side effects remain poorly understood. This Research Topic highlights research that creates new knowledge of cellular and molecular signaling pathways using preclinical animal models. To address the impact of genetic bases and disparate responses to RT, Choudens et al. used Dahl salt-sensitive rats as a surrogate hypertension model to analyse the injury to the heart by irradiation. Similarly using a rat model of lung irradiation, Wiedemann et al. demonstrated a series of pathologies following remodeling of the pulmonary vasculature. The similarities between the mechanisms of vascular remodeling in pulmonary hypertension and those after irradiation could be translated into interventions that benefit patients treated for thoracic tumors, where radiation to lung tissue often cannot be avoided. This knowledge greatly facilitates the discovery of biomarkers for cardiovascular toxicity, the identification of new cardioprotective therapeutic targets, and the optimization of prevention and intervention strategies in chemo- and RT. Cancer immune checkpoint inhibitors have led to recent advances in the field of cancer immunotherapy improving overall survival in multiple cancers with historically abysmal prognoses. Cardiac-specific immune-related adverse events are potentially fatal. However, the understanding of autoimmune cardiotoxicity remains limited due to its rareness. Irabor et al. provide a literature review on the pathologic mechanisms, diagnosis, and management of autoimmune cardiotoxicity resulting from ICIs and offer a perspective on potential strategies and research to prevent and mitigate their occurrence. Thoracic RT has been associated with increased cardiac morbidity and mortality in numerous studies, including the landmark Darby´s study (2013), demonstrating a linear increase in cardiac mortality with increasing mean cardiac radiation dose. However, the extent to which cardiotoxicity has been incorporated as an endpoint in RT studies has not been standardized and is sometimes unknown. To better characterize cardiac toxicities, future prospective studies involving thoracic RT should include cardiotoxicity endpoints as recommended in the study by Prasad et al.. We hope that this Research Topic will arouse the interest of radiation researchers, epidemiologists and clinicians to continue to pursue research that increases our knowledge on CVD following chemo- and RT, eventually implementing practices that will improve the safety of cancer therapy.

## Authors contributions

All authors listed have contributed equally. VM drafted the manuscript and incorporated the ideas of all authors. MB, OA and NP provided comments and approved of the final version.

## Acknowledgments

The authors would like to thank all scientists who contributed to this Research Topic as well as various reviewers/editors of the respective manuscripts, for their efforts, timely responses and enthusiasm. We also thank the Frontiers Editorial Office for their assistance and support.

## Conflict of interest

The authors declare that the research was conducted in the absence of any commercial or financial relationships that could be construed as a potential conflict of interest.

## Publisher’s note

All claims expressed in this article are solely those of the authors and do not necessarily represent those of their affiliated organizations, or those of the publisher, the editors and the reviewers. Any product that may be evaluated in this article, or claim that may be made by its manufacturer, is not guaranteed or endorsed by the publisher.

